# Phthalate Exposure During Pregnancy and Lactation Transgenerationally Impairs the Epididymis in the Offspring of Rats

**DOI:** 10.1002/jbt.70379

**Published:** 2025-06-23

**Authors:** Ana Paula Franco Punhagui‐Umbelino, Giovanna Fachetti Frigoli, Ariana Musa de Aquino, Barbara Campos Jorge, Luiz Guilherme Alonso‐Costa, Rafaela Pires Erthal‐Michelato, Arielle Cristina Arena, Wellerson Rodrigo Scarano, Glaura Scantamburlo Alves Fernandes

**Affiliations:** ^1^ Department of General Biology, Biological Sciences Center State University of Londrina Londrina Brazil; ^2^ Department of General Pathology, Biological Sciences Center State University of Londrina Londrina Brazil; ^3^ Morphology Department, Institute of Biosciences Universidade Estadual Paulista Júlio de Mesquita Filho, UNESP Botucatu Brazil

**Keywords:** epididymis, gene expression, phthalate esters, plasticizers, pollution, toxicity

## Abstract

Phthalates are chemical compounds used as plasticizers in plastic products, for guaranteeing their flexibility. These compounds damage the epididymal morphophysiology. This study aimed to verify the changes in the epididymis of neonatal and adult rats over two generations. Pregnant Sprague–Dawley females were divided into four groups and gavaged daily from GD10 to PND21 with corn oil (Control:C) or the phthalate mixture at three doses (20, 200, and 200 mg/Kg). F1 animals were euthanized at 22PND and 120PND; unexposed females were mated with 90PND males to obtain the F2 generation. Male rats were euthanized at 22PND. Tissue remodeling of the compartments was observed at all doses in the 22PND animals of both generations. Treatment at 200 mg/Kg resulted in alterations, a decrease in the epithelium and a consequent increase in the lumen in the cauda in F1 PND22. The lumen in the caput and cauda increased, with a reduction in the interstitium at 200 mg/Kg in F2 PND22. The lumen diameter was reduced in the caput and increased in the interstitium at the two highest doses in adult animals. The expression of *GPR30*, *GPX3*, *GSR*, *IL10*, and *TNFa* was reduced in adult animals at the highest dose. *GPR30* expression increased at 200 mg/Kg dose in PND22. *TNFa* expression was reduced at all doses in F1 PND22 animals and significantly increased in F2 PND22 animals at 200 µg/Kg and 200 mg/Kg doses. In conclusion, phthalates modify the epididymal structure and impair gene expression, mainly in the late phase.

## Graphical abstract

This study reveals that phthalate exposure induces tissue remodeling and alters gene expression in the epididymis of Sprague‐Dawley rats across two generations. The findings underscore the transgenerational impact of phthalates, highlighting their influence on oxidative stress and inflammatory pathways during critical developmental windows.

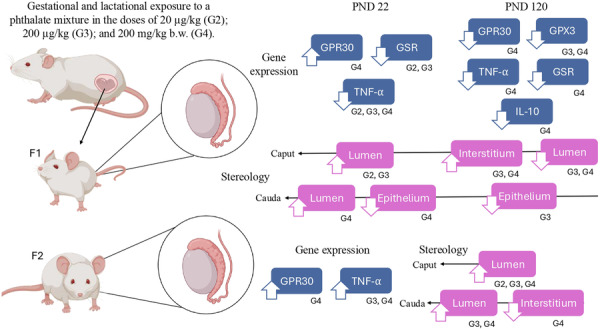

## 1 Introduction

The Developmental Origins of Health and Disease (DOHaD) hypothesis posits that environmental factors during early life can predispose individuals to diseases later in life. A mismatch between predicted and actual developmental environments may negatively impact health and increase disease risk [1]. During the developmental phase, organisms exhibit heightened plasticity, making gene expression and cellular signaling pathways more vulnerable to external influences, including environmental contaminants [2].

Phthalate esters, commonly used as plasticizers, enhance the flexibility and durability of various products, including children's toys, automotive parts, and household items [3]. However, during heating and cooling, plastic containers can leach phthalates, contaminating food and beverages [4].

Exposure to phthalates can lead to adverse effects on male reproductive function at any age, manifesting immediately, later in life, and even across generations [3, 4]. Phthalates are detectable in the blood and urine of pregnant and lactating women, raising concerns about potential physiological changes in newborns, which suggest transgenerational effects [5]. Research by Parks et al. [6] demonstrated that male rats exposed to phthalate esters during gestation and lactation experienced Leydig cell hyperplasia and dysfunction in sexual differentiation, along with reduced testosterone synthesis. Furthermore, exposure to varying doses of phthalates during pregnancy has been associated with increased levels of leukemia inhibitory factor mRNA (LIF) and altered testicular testosterone and steroidogenic capacity [7]. These changes correlate with a rise in abnormal seminiferous tubules and a higher incidence of male genital tract malformations [8].

In vitro studies with MEHP phthalate showed no alterations in testosterone levels within seminiferous tubules, but significant changes were noted in germ cells [9]. This contrasts with human studies that indicate testosterone inhibition without changes in Leydig cell numbers, along with increased apoptosis in Sertoli cells and imbalances in germ cell proportions during spermatogenesis [10].

In experiments involving adult male rats exposed to different doses of DBP, researchers observed dose‐dependent epididymal toxicity characterized by decreased glutathione peroxidase (GSH‐Px) and superoxide dismutase (SOD) activity, alongside elevated malondialdehyde (MDA) levels. Additionally, there were reports of epididymal tubule atrophy and vascular hyperemia [11], indicating significant damage to the male reproductive system [12].

Estrogen is transported into main and interstitial cells of the epididymis via the GPR30‐encoded G‐protein coupled receptor, leading to inflammation influenced by phthalates, which disrupt ionic balance in cells [13]. Antioxidant actions are mediated by GPX3 and GSR via glutathione, while pro‐inflammatory proteins are encoded by IL10 and TNF‐α [14].

Overall, phthalate exposure at any life stage—whether direct or indirect—shows a positive correlation with reproductive damage during spermatogenesis and sperm capacitation, producing immediate and transgenerational effects on development. This study aims to assess epididymal parameters in rats exposed to a mixture of phthalate esters during gestation and lactation across two generations (F1 and F2).

## 2 Materials and Methods

### 2.1 Animals

Adult males (*n* = 15; 90 days postnatal—PND) and adult females (*n* = 50; PND120) of the Sprangue–Dawley strain from the Multidisciplinary Center for Biological Research in Laboratory Animal Science (CEMIB/UNICAMP) were kept in the Small Mammalian Animal Facility of the Morphology Department of IBB/UNESP under controlled light conditions (12 L, 12D photoperiod, lights switched off at 6:00 p. m.) and temperature (23°C ± 2°C) with water and feed ad libitum. All experimental procedures and protocols complied with the Ethical Principles of Animal Research, adopted by the Brazilian College of Animal Experimentation, and were approved by the Ethics Committee on the Use of Animals (CEUA) of the Biosciences Institute of UNESP de Botucatu (Protocol 1040/CEUA).

### 2.2 Experimental Design

Pregnant female rats were randomly divided into 4 experimental groups (*n* = 10/group), all treated by gavage: Group 1: control (vehicle: corn oil); Group 2: 20 µg/Kg/day of the mixture of phthalates diluted in corn oil; Group 3: 200 µg/Kg/day of the mixture of phthalates diluted in corn oil; Group 4: 200 mg/Kg/day of the mixture of phthalates diluted in corn oil. The mixture of phthalates was in the following ratio: 19% DEHP (Bis(2‐ethylhexyl) phthalate), 36% DEP (Diethylphthalate), 15% DBP (Di‐n‐butylphthalate), 10% DiBP (DiisobutylPhthalate), 8% BBzP (Butylbenzylphthalate), and 10% DiNP (Diisononylphthalate); based on the composition of phthalates detected in urine samples from pregnant women in Illinois, USA [15]. Treatment in pregnant rats occurred from gestational Day 10 (GD10) to postnatal Day 21 (PND21), a critical period for the development of the urogenital apparatus [16, 17]. The female rats were kept in individual cages, being weighed on alternate days for the calculation of the volume of the phthalate mixture to be administered and the investigation of clinical signs of toxicity [18].

*F1 generation:* After birth, the number of pups was reduced to eight pups per rat, in a 1:1 ratio between females and males, determined by the year‐genital distance (AGD) [19]. At PND22 five male rats from each group were anesthetized and euthanized, while the remaining animals remained until adulthood (PND 120) to obtain the F2.

*F2 generation:* At PND90, five male rats from each group (n = 20) of the F1 generation obtained were mated with untreated virgin female rats of the Sprangue‐Dawley strain (PND 120) with the same protocol as above. The F1 generation males were kept until PND 120, then they were anesthetized and euthanized. F2 offspring were kept until weaning (PND22) and then anesthetized and euthanized.

**Figure 1:**
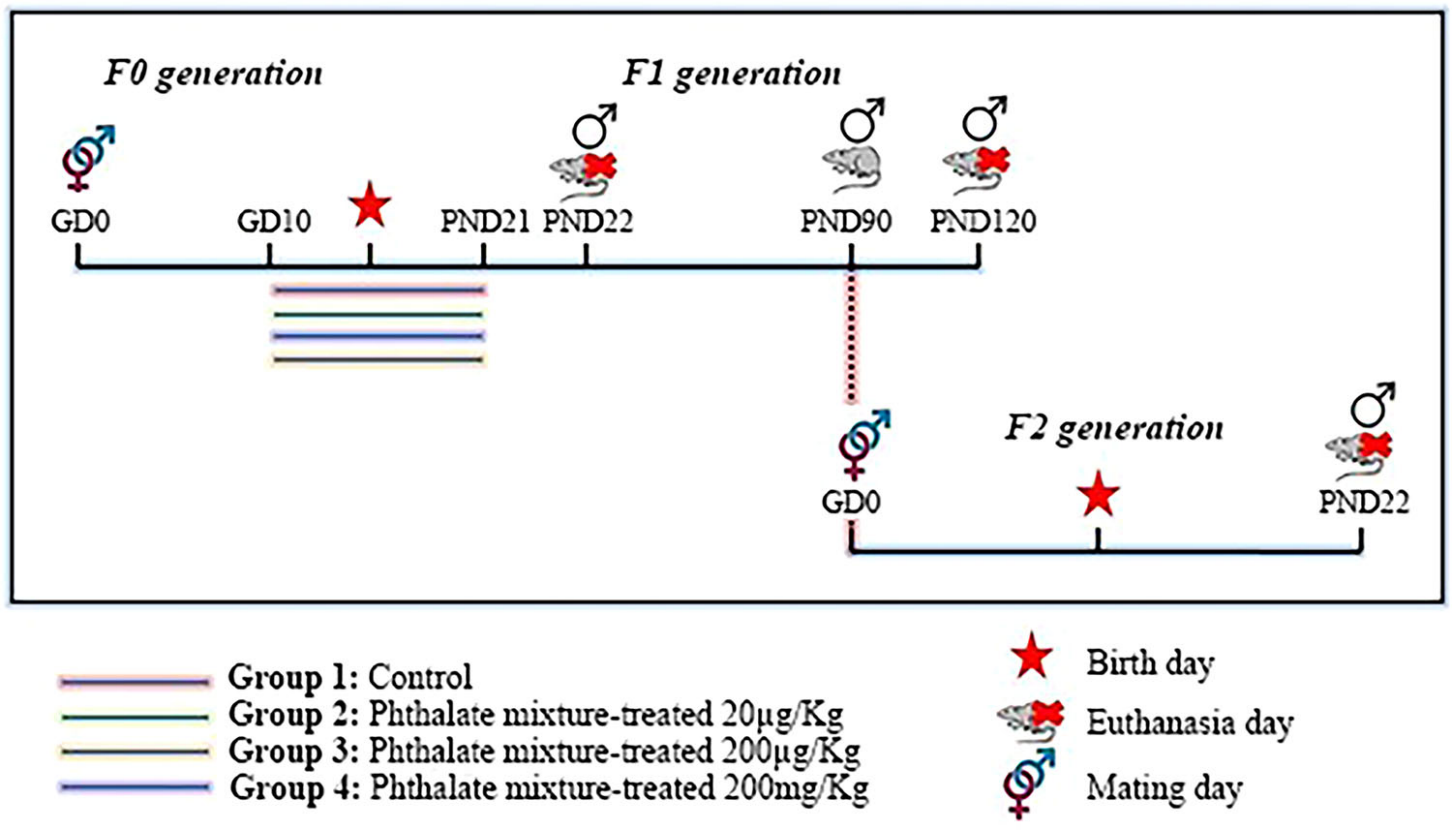
Experimental design of the study. *GD*, gestational day; *PND*, postnatal day.

The epididymis of the animals were collected to obtain their weight. The left epididymis of all animals was used for histological analyses of stereology. The right epididymis was used for evaluation of mRNA targets by RT1‐PCR, in which it was possible to verify molecular alterations. The experimental design is summarized in Figure 1.

### 2.3 Histological Process

The left epididymis (5/group) were removed and fixed in Methacarn (60% Methanol, 30% Chloroform and 10% Glacial Acetic Acid). This material was then immersed in a few baths (20 min each) of alcohol (95% and absolute), xylene (40 min) and Paraplast. The last xylene and Paraplast baths were performed in an oven at 65°C, after which the material was sectioned into 5 µm semi‐serial slices per animal and stained with hematoxylin and eosin (HE) [20].

#### 2.3.1 Epididymal Stereology

Ten random epididymal cross‐sections from the initial segment 2 A and 5 A/B regions were obtained per adult animal. The epididymis of neonates were analyzed without division into regions. This analysis was performed using Weibel's multipurpose graticulate, with 168 points, to compare the relative proportion among the epididymal components (epithelium, stroma, and lumen) in the experimental groups [20].

### 2.4 Evaluation of Gene Expression by Real‐Time Polymerase Chain Reaction After Reverse Transcription (Rt‐qPCR): GPR30, GPX3, GSR, IL10 and TNF

Gene expression in epididymis cells was evaluated by RT‐qPCR related to testosterone conversion (*GPR30* gene), inflammation (*IL10* and *TNF* genes), and oxidative stress of glutathione (*GPX3* and *GSR* genes). RNA extraction was performed with the Trizol kit (Ambion, USA) according to the manufacturer's instructions. RNA was quantified by spectrophotometry using the NanoVue equipment (GE Healthcare Life Sciences, USA). The analysis of RNA quality was obtained by the RNA integrity number (RNA Integrity Number, RIN), from the analysis of ribosomal RNAs based on microfluids, using the 2100 Bioanalyzer system (Agilent, USA) [21, 22]. The mRNA Reverse Transcription Reaction was performed using the High‐Capacity RNA‐to‐cDNA Master Mix kit (Life Technologies, USA), following the manufacturer's guidelines. For the reaction, 4 μL of Master Mix for reverse transcription was used, to which 1 μg of RNA was added and the volume made up to 20 μL with nuclease‐free water. The mixture was incubated under the following conditions: 25°C for 5 min., 42°C for 30 min. followed by reverse transcriptase inactivation at 85°C for 5 min. For each RT‐qPCR reaction for mRNAs, 10 μL of GoTaq qPCR Master Mix, based on SYBR Green chemistry (Promega, USA), 5 μL of the RT reaction and 1 μL of “sense” and “antisense” primers were used at 10 μM and the volume was made up to 20 μL with nuclease‐free water. Primers for the genes were designed using the Primer‐Blast program (http://www.ncbi.nlm.nih.gov/tools/primer-blast/), present in Table 1. Thermocycling was performed in the QuantStudio equipment (Life Technologies, USA), under the following conditions: GoTag Hot Start Polymerase activation 2 min. at 95°C followed by 40 cycles of 15 s. at 95°C and 1 min. at 60°C, finally, a dissociation curve in the range of 60°C–95°C. An amplification graph was plotted for each sample showing an increase in fluorescent reporter dye (ΔRn) in each PCR cycle. From this graph, the cycle where the reaction crosses the detection threshold (cycle threshold—CT) was determined. The relative quantification of each gene was performed using the 2‐ΔΔ CT method according to Livak and Schmittgen [23]. The values obtained for all samples were normalized by the ratio obtained between the target genes and the endogenous *GAPDH* gene.

**Table 1:** The target epididymal genes and the GAPDH endogenous gene, forward and reverse gene sequence, and the ID number for all genes.

Gene	ID number	Foward sequence	Reverse sequence
GPX3	NM_022525.4	CTTCTCACCCCAGTTGCGAT	AGTCTATGGGCGAGTTTCCG
GSR	NM_053906.2	CGGAAACTCGCCCATAGACTT	ATGGACGGCTTCATCTTCAGT
IL‐10	NM_012854.2	TGCGACGCTGTCATCGATTT	TGGCCTTGTAGACACCTTTGT
TNF	L19123.1	GGTCTCATCTCCGCCTTTGT	CCCAAAATCCTGCCCTGTCA
GPR30	NM_133573.2	CCTGCCGACTTCGCAAGT	CCACACCGTTCTCTCCTGGAT
Gapdh	NM_017008.4	GCTCTCTGCTCCTCCCTGTTC	GAGGCTGGCACTGCACAA

### 2.5 Statistical Analysis

One‐way analysis of variance (ANOVA) with post hoc Dunnett's test, mean ± SD, or the non‐parametric Kruskal–Wallis test with post hoc Dunn's test was used, median (Q1–Q3), depending on the data distribution, to compare the results between the phthalate‐treated groups and the control group. The variance among the experimental groups was compared using Bartlett's test. The Shapiro–Wilk test was performed to evaluate the normal distribution. When required, the data were normalized via box‐cox transformation. Differences were considered significant when *p* < 0.05. The statistical analyses and graph design for the results were performed using GraphPad Prism for Windows (version 7.01—GraphPad Software, La Jolla, California, USA) and Instat (version 3.01—GraphPad Software, La Jolla, California, USA) programs.

## 3 Results

### 3.1 Body and Reproductive Organ Weight

The body and absolute epididymal weights were similar among all groups and animals (Table 2).

**Table 2:** Body and epididymis weight.

	Control (*n* = 5)	20 µg/Kg (*n* = 5)	200 µg/Kg (*n* = 5)	200 mg/Kg (*n* = 5)
F1 22DPN				
Body weight (g)	48.760 ± 3.322	48.360 ± 4.525	47.830 ± 3.426	46.460 ± 8.473
Epididymis weight (g)	0.015 ± 0.003	0.016 ± 0.003	0.015 ± 0.001	0.014 ± 0.004
F1 120DPN				
Body weight (g)	469.7 ± 13.10	432.7 ± 57.86	428.1 ± 24.40	481.3 ± 65.51
Epididymis weight (g)	0.567 ± 0.044	0.534 ± 0.115	0.601 ± 0.028	0.599 ± 0.034
F2 22DPN				
Body weight (g)	42.18 ± 4.57	41.71 ± 5.33	44.56 ± 6.34	35.56 ± 3.13
Epididymis weight (g)	0.013 ± 0.003	0.011 ± 0.003	0.014 ± 0.001	0.011 ± 0.002

*Note:* Mean ± Standard Deviation; ANOVA *post test* Dunnett.

### 3.2 Epididymal Stereology

The luminal compartment in the caput region was increased in the F1 PND22 animals at doses of 20 and 200 µg/Kg, while in the cauda region there was a decrease in the epithelium and consequent increase in the lumen at the dose of 200 mg/Kg. In addition, there was an isolated increase in the luminal compartment at a dose of 20 µg/Kg. In adult animals of the F1 generation, an increase in the interstitium was observed in the caput region with a consequent decrease in the lumen at 200 µg/Kg and 200 mg/Kg; in the cauda, there was an isolated decrease in the epithelium at the dose of 200 µg/Kg (Table 3).

**Table 3:** Epididymal stereology.

		Control (*n* = 5)	20 µg/Kg (*n* = 5)	200 µg/Kg (*n* = 5)	200 mg/Kg (*n* = 5)
F1 22DPN	**Caput**				
	Epithelium	33.93 (24.11–38.69)	30.95 (24.70–36.31)	31.55 (19.64–36.76)	27.38 (22.62–34.52)
	Interstitium	60.12 (54.17–68.01)	58.93 (51.49–60.71)	57.14 (49.55–65.03)	59.23 (54.32–66.07)
	Lumen	6.84 (4.76–9.97)	10.71 (7.14–15.92)*	11.90 (8.92–14.73)*	11.31 (7.14–14.88)*
	**Cauda**				
	Epithelium	27.68 (21.73–34.52)	28.57 (23.21–34.52)	27.38 (18.75–33.78)	19.35 (15.48–24.26)*
	Interstitium	62.20 (55.06–70.39)	58.93 (52.98–63.10)	63.10 (56.40–71.43)	64.88 (59.67–66.96)
	Lumen	7.73 (5.80–10.71)	11.61 (7.58–16.52)*	8.33 (5.95–10.57)	15.77 (12.50–19.64)*
F1 120DPN	**Caput**				
	Epithelium	20.83 (18.45–23.81)	21.73 (19.05–23.81)	20.83 (18.15–25.60)	20.83 (18.75–24.70)
	Interstitium	13.69 (9.67–19.49)	14.29 (11.31–21.13)	25.60 (16.07–32.14)*	19.05 (14.88–26.49)*
	Lumen	65.18 (57.29–70.98)	62.20 (54.02–68.30)	55.36 (38.39–65.48)*	60.71 (50.60–64.29)*
	**Cauda**				
	Epithelium	16.07 (11.01–19.79)	12.50 (8.78–16.22)	6.54 (3.27–12.20)*	14.88 (9.52–19.49)
	Interstitium	18.15 (10.71–22.17)	19.35 (15.33–26.19)	17.26 (9.82–25.00)	20.54 (14.43–25.45)
	Lumen	67.56 (60.57–76.34)	66.37 (61.16–75.00)	72.62 (64.58–84.52)	65.18 (58.04–72.47)
F1 22DPN	**Caput**				
	Epithelium	32.44 (24.70–36.90)	30.36 (25.60–35.42)	31.55 (20.68–36.31)	27.98 (22.62–32.59)
	Interstitium	58.33 (54.17–65.48)	58.63 (52.83–60.71)	58.04 (51.19–63.24)	59.52 (55.36–65.03)
	Lumen	8.92 (5.95–10.71)	10.71 (7.14–15.03)*	11.90 (9.37–14.29)*	11.31 (8.63–14.43)*
	**Cauda**				
	Epithelium	32.44 (24.70–36.90)	30.36 (25.60–35.42)	31.55 (20.68–36.31)	27.98 (22.62–32.59)
	Interstitium	64.58 (58.18–69.64)	62.20 (55.06–70.39)	64.88 (59.67–66.96)	58.93 (52.98–63.10)*
	Lumen	8.33 (6.54–10.12)	7.73 (5.80–10.71)	15.77 (12.50–19.64)*	11.61 (7.58–16.52)*

*Note:* Median (Q1–Q3), Kruskall–Wallis *post test* Dunn.

* Indicates groups that differ from the control. It is considered a statistical difference when *p* < 0.05.

**Figure 2:**
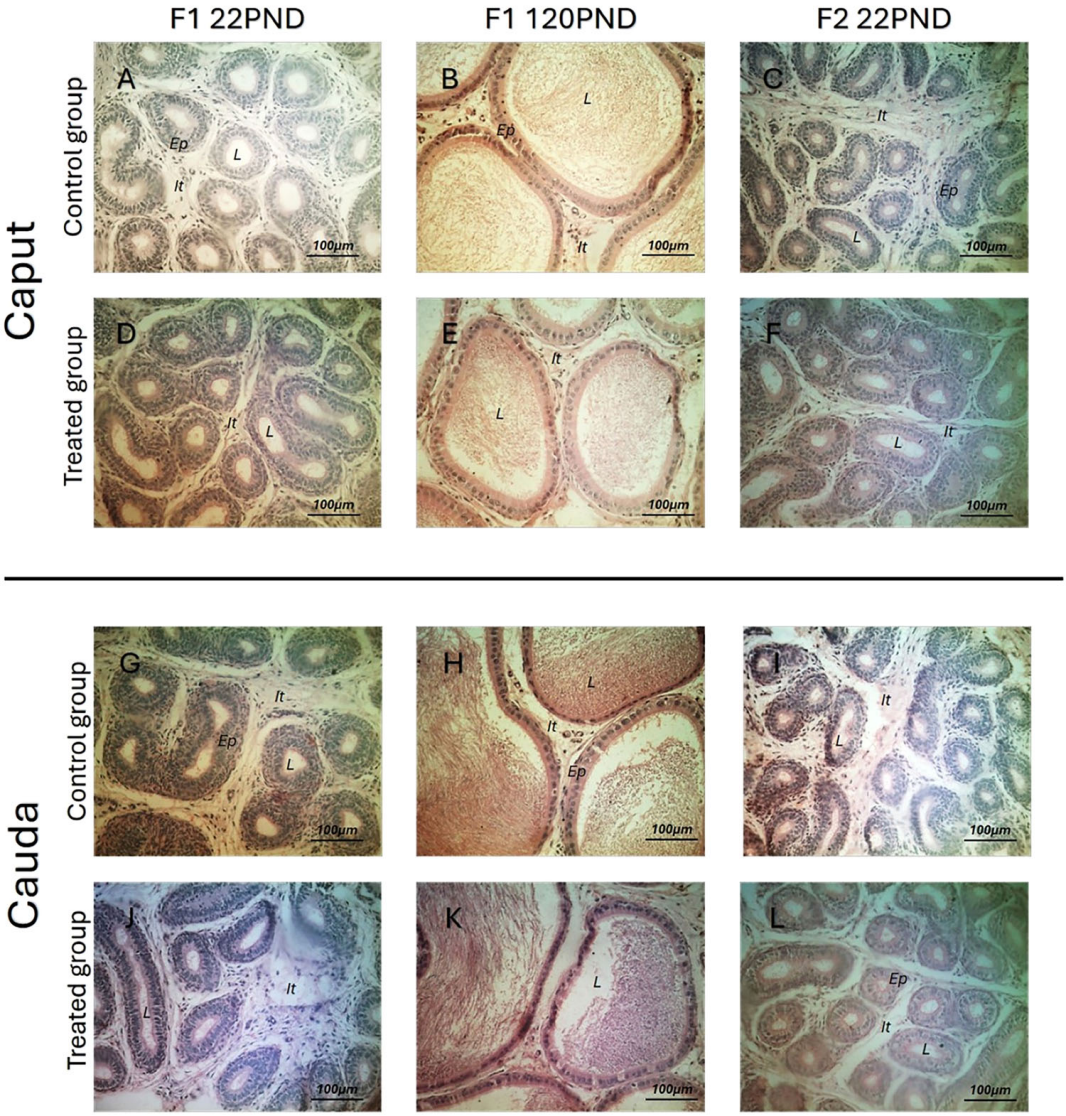
Histological photomicrographs of the epididymal tissue. A, B, C, G, H, I: control group; D, E, F, J, K, L: phthalate groups; A, D, G, J: F1 22PND animals; B, E, H, K: F1 120PND animals; C, F, I, L: F2 22PND animals.

In the F2 generation pups, there was an increase in the lumen in the caput at all doses of the phthalate mixture, whereas in the cauda, there was an increase in the lumen at doses of 200 µg/Kg and 200 mg/Kg, and a consequent decrease in the interstitial compartment only at a dose of 200 mg/Kg (Table 3). Epididymal histology of the experimental groups is demonstrated in Figure 2.

### 3.3 Gene Expression Analysis Using RT‐qPCR

**Figure 3:**
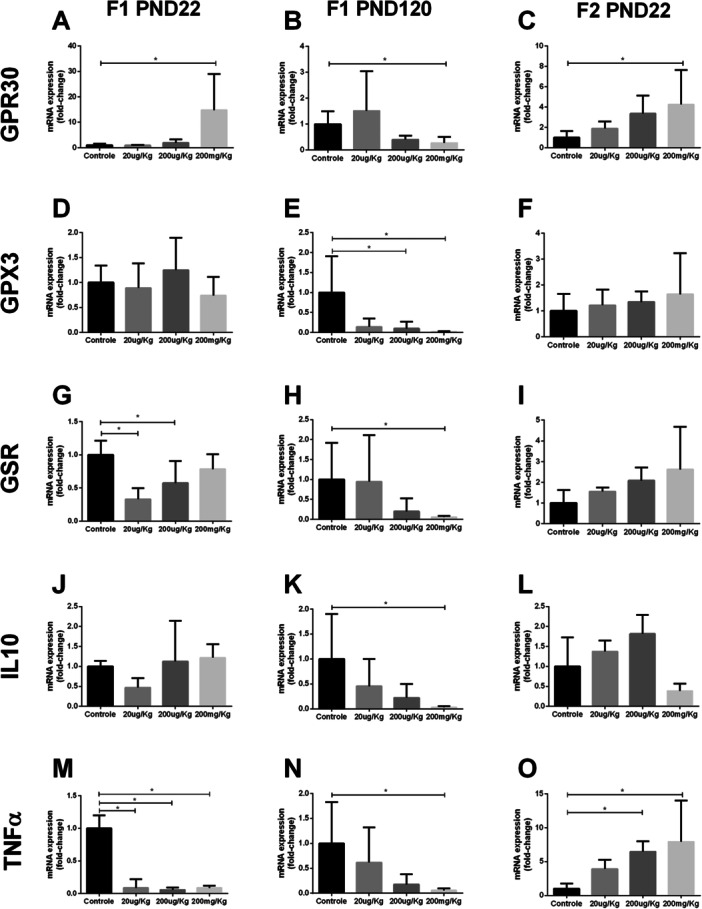
Expression of *GPR30* (A, B, C), *GPX3* (D, E, F), *GSR* (G, H, I), *IL10* (J, K, L), and *TNFa* (M, N, O) in epididymal tissue. Data are expressed as mean ± SEM (n = 5); One‐way ANOVA with Dunnett's post hoc test. * Indicates groups that differ significantly from the control group (*p* < 0.05).

GPR30 expression was increased in pups of both generations (Figure 3A,C), whereas it was reduced in adult animals at the same dose of 200 mg/Kg (Figure 3B).

GPX3 gene expression decreased significantly only in adult animals at doses of 200 µg/Kg and 200 mg/Kg (Figure 3E); however, in infants, it was similar to that in the controls (Figure 3D,F). The expression of glutathione receptor (GSR) decreased in F1 animals at doses of 20 and 200 µg/Kg in pups (Figure 3G) and only at a dose of 200 mg/Kg in adults (Figure 3H); the expression was similar between groups in the F2 generation (Figure 3I).

The expression of inflammatory mediators such as IL10 decreased in adult animals at a dose of 200 mg/Kg (Figure 3K), while no significant changes were observed in the infants (Figures 3J and 2L). The expression of TNF‐α decreased at all doses in F1 PND22 animals (Figure 3M); however, it was reduced only at the dose of 200 mg/Kg in F1 PND120 animals (Figure 3N); it was significantly increased at doses of 200 µg/Kg and 200 mg/Kg in F2 PND22 animals (Figure 3O).

## 4 Discussion

A mixture of phthalate esters adversely affected epididymal tissue, with each dosage impacting various tissue proportions and causing remodeling alongside immediate, delayed, and transgenerational effects. Changes in gene expression were observed specifically in GPR30, GSR, and TNF‐α, highlighting the influence of phthalates on the oxidative and inflammatory characteristics of the epididymis in postnatal day 22 (PND22) animals. Notably, GPR30 expression increased, GSR expression decreased, and TNF‐α expression varied—decreasing in the F1 generation and increasing in the F2 generation. In contrast, in adult animals, only the 200 µg dose led to significant remodeling of the epididymal tissue, characterized by an enlarged interstitial lumen and reduced caput region lumen. The cauda of these animals exhibited greater resilience to phthalate exposure. This study confirmed that phthalates have transgenerational effects and that the blood‐epididymal barrier fails to prevent phthalate‐induced damage.

The male reproductive system comprises numerous receptors that respond to hormonal and metabolic fluctuations. One of the most crucial is the G protein‐coupled receptor 30 (GPR30) [4]. This transmembrane protein facilitates the uptake of estrogen via a nonclassical pathway. Although its role in epididymal tissue remains poorly understood, diminished regulation of GPR30 can compromise duct structure [24]. The highest dosage of the phthalate mixture significantly increased GPR30 expression in PND22 animals while decreasing it in adults. This finding elucidates the receptor's role in tissue remodeling throughout the spermatogenesis cycle and the overall functionality of the epididymis.

The prenatal development phase is critical for the formation of various structures and organs, with final maturation occurring postnatally when all proteins and receptors are expressed. Any disruption during this period can lead to immediate dysfunctions or even transgenerational defects [25, 26, 27]. Reduced expression of genes associated with development, differentiation, and oncogenesis has been linked to lower serum testosterone levels at the lowest dose of the phthalate mixture in neonatal rats [18]. Consequently, epididymal tissues are particularly sensitive to phthalate mixtures, which induce tissue remodeling.

Research on urine samples from pregnant women indicates varying doses of phthalates acquired through environmental and daily exposure to phthalate‐containing products. These phthalates have been shown to increase the expression of inflammatory and oxidative stress biomarkers, demonstrating their toxicity and linking their presence to heightened inflammation [28]. While GPX3 expression did not immediately affect F1 PND22 animals, it reduced the late glutathione response in F1 PND120 animals exposed to both 200 µg and 200 mg doses. Conversely, GSR expression in F1 PND22 animals decreased immediately at the lowest dose, dropped further at the highest dose in adult animals, and remained unchanged across generations. This suggests that oxidative stress directly affects the generation exposed to phthalates, as subsequent generations are exposed through mothers during pregnancy and lactation.

Toxicological studies have shown that lower doses can result in more significant tissue damage because the body accumulates small amounts of toxicants over time, while higher doses are often metabolized and excreted more efficiently [18, 29, 30]. The epididymis has a blood‐epididymal barrier that ensures a specialized luminal microenvironment for sperm maintenance, restricting the passage of ions and macromolecules during prenatal development and puberty remodeling [31, 32]. However, this barrier is still maturing in prepubertal animals, allowing all phthalate doses to potentially alter the proportions of epididymal compartments. In adult animals, where spermatogenesis is complete, higher doses of phthalate mixtures can disrupt tissue organization.

An increase in inflammatory mediators signals the body's response to foreign substances, indicating an acute inflammatory process that involves pro‐inflammatory cytokine production [33]. In vitro studies and research involving mice demonstrate that exposure to various phthalates elevates the production of pro‐inflammatory cytokines like IL‐1β [34] and IL‐6 [35], leading to reduced testosterone production and increased macrophage recruitment [36]. Interleukin‐10, an anti‐inflammatory cytokine, plays a vital role in preventing inflammatory and autoimmune diseases; however, under certain conditions, it can exacerbate local inflammation and related symptoms. Elevated IL‐10 levels can impair host responses to microbial threats and hinder recovery from associated tissue damage and hemodynamic disturbances [37].

Epidemiological studies support a link between phthalates and inflammation. Higher urinary concentrations of phthalate metabolites in allergic conditions have been associated with increased immune responses in both experimental and clinical contexts [38]. Trim et al. [39] noted that inflammation did not correlate with phthalate exposure, as no inflammatory cytokines were positively identified in urinary samples from exposed patients. This study revealed that phthalates can reduce the expression of pro‐inflammatory cytokines; TNF‐α expression showed immediate alterations but remained reduced only at the highest phthalate dose in adult animals. The observed increase in expression at higher phthalate doses suggests a strong relationship between phthalate presence and inflammation in F2 animals.

## 5 Conclusion

The postnatal period is an important developmental window, and any disturbance to the internal maternal environment causes harmful effects on the offspring. The prepubertal animals of the F1 and F2 generations were more sensitive to the action of phthalates than adult animals that had already achieved immunity maturation. In addition, we observed the transgenerational effects of phthalates; the alterations in gene expression were observed in the next generation of animals derived from those previously exposed to environmental toxicants.

## Author Contributions

**Ana Paula Franco Punhagui‐Umbelino:** conceptualization, investigation, writing – original draft, methodology, validation, visualization, writing – review and editing, software, formal analysis, data curation. **Giovanna Fachetti Frigoli:** conceptualization, investigation, methodology, writing – review and editing. **Ariana Musa de Aquino:** conceptualization, investigation, formal analysis, data curation, methodology, validation. **Barbara Campos Jorge:** conceptualization, investigation, methodology. **Luiz Guilherme Alonso‐Costa:** conceptualization, investigation, methodology. **Rafaela Pires Erthal‐Michelato:** conceptualization, investigation, methodology, data curation, formal analysis. **Arielle Cristina Arena:** conceptualization, investigation, funding acquisition, writing – review and editing, resources. **Wellerson Rodrigo Scarano:** conceptualization, investigation, funding acquisition, writing – review and editing, resources, project administration. **Glaura Scantamburlo Alves Fernandes:** conceptualization, investigation, funding acquisition, writing – review and editing, project administration, supervision, resources.

## Conflicts of Interest

The authors declare no conflicts of interest.

## Data Availability

All data supporting the findings of this study are available within the paper.
